# Evaluation of cell death pathways initiated by antitumor drugs melatonin and valproic acid in bladder cancer cells

**DOI:** 10.1002/2211-5463.12223

**Published:** 2017-04-27

**Authors:** Siwei Liu, Bilin Liang, Huiting Jia, Yuhan Jiao, Zhongqiu Pang, Yongye Huang

**Affiliations:** ^1^ College of Life and Health SciencesNortheastern UniversityShenyangChina

**Keywords:** apoptosis, autophagy, epithelial–mesenchymal transition, ER stress, melatonin, valproic acid

## Abstract

Effective drug combinations have the potential to strengthen therapeutic efficacy and combat drug resistance. Both melatonin and valproic acid (VPA) exhibit antitumor activities in various cancer cells. The aim of this study was to evaluate the cell death pathways initiated by anticancer combinatorial effects of melatonin and VPA in bladder cancer cells. The results demonstrated that the combination of melatonin and VPA leads to significant synergistic growth inhibition of UC3 bladder cancer cells. Gene expression studies revealed that cotreatment with melatonin and VPA triggered the up‐regulation of certain genes related to apoptosis (TNFRSF10A and TNFRSF10B), autophagy (BECN, ATG3 and ATG5) and necrosis (MLKL, PARP‐1 and RIPK1). The combinatorial treatment increased the expression of endoplasmic reticulum (ER)‐stress‐related genes ATF6, IRE1, EDEM1 and ERdj4. Cotreatment with melatonin and VPA enhanced the expression of E‐cadherin, and decreased the expression of *N*‐cadherin, Fibronectin, Snail and Slug. Furthermore, the Wnt pathway and Raf/MEK/ERK pathway were activated by combinatorial treatment. However, the effects on the expression of certain genes were not further enhanced in cells following combinatorial treatment in comparison to individual treatment of melatonin or VPA. In summary, these findings provided evidence that cotreatment with melatonin and VPA exerted increased cytotoxicity by regulating cell death pathways in UC3 bladder cancer cells, but the clinical significance of combinatorial treatment still needs to be further exploited.

**Abbreviations**
APC: adenomatous polyposis coli
ATF6: activating transcription factor 6
ATG3: autophagy‐related gene 3
ATG5: autophagy‐related gene 5
BECN: beclin 1
EDEM1: ER degradation enhancing alpha‐mannosidase like protein 1
ERdj4: endoplasmic reticulum DnaJ homolog 4
FAS: Fas cell surface death receptor
HDAC: histone deacetylase
HRK: harakiri, BCL2‐interacting protein
IRE1: inositol‐requiring enzyme 1
LC3: microtubule‐associated protein 1 light chain 3
LEF1: lymphoid enhancer‐binding factor 1
MLKL: mixed lineage kinase domain‐like
PARP‐1: poly (ADP‐ribose) polymerase 1
RIPK1: receptor activated protein kinase 1
TNFRSF10A: TNF‐receptor superfamily member 10A
TNFRSF10B: TNF‐receptor superfamily member 10B
VPA: valproic acid
Wnt3a: Wnt family member 3A
Wnt5a: Wnt family member 5A
XBP1: X‐box‐binding protein 1

Bladder cancer is one of the most common malignancies worldwide, with 74 000 new cases diagnosed and 16 000 deaths in 2015 1. The occurrence of bladder cancer is strongly associated with exposure to environmental factors, and cigarette smoking is considered the single most crucial environmental factor for determining risk 2. Surgical resection, intravesical therapy, chemotherapy and radiotherapy are the main therapeutic methods. Despite many therapeutic advances over the last decade, the mortality of bladder cancer has not substantially improved. Therefore, the development of novel treatment is still needed.

Melatonin (*N*‐acetyl 5‐methoxytryptamine), the main hormone secreted from the pineal gland, is a well‐known antioxidant and free radical scavenger with protective effects against oxidative damage in several tissues 3. Besides being a potent antioxidant, melatonin has attracted more and more attention as a potential natural oncostatic agent. Studies have shown that melatonin has the potential of being used as a therapeutic agent for treating various types of human cancer, such as breast cancer 4, colon cancer 5, ovarian cancer 6, prostate cancer 7 and lung cancer 8. Melatonin exhibits various pharmacological effects against cancer, including suppression of the expression of matrix metalloproteinases 9, 10 and inhibition of invasion and metastasis of cancer cells 11, 12. Melatonin suppresses MMP‐9 transcription via modulating the expression of CREB‐binding protein (CREBBP) and E1A‐binding protein p300 (EP300), and decreasing histone acetylation 13, suggesting that histone acetylation plays important roles in melatonin‐related cancer treatment. In fact, many recent studies show that histone acetylation was induced during melatonin treatment 14, 15, 16, 17, 18. In addition, inhibition of histone deacetylase (HDAC) signaling sensitized cancer cells to melatonin treatment 19, indicating that melatonin in combination with HDAC inhibitors may be a potential therapeutic intervention for human cancer.

Histone deacetylase inhibitors show anticancer effects via cell‐cycle arrest, differentiation induce and increased apoptosis in various cancer cell types, including bladder cancer cells 20, 21, 22. Valproic acid (VPA, 2‐propylpentanoic acid), an HDAC inhibitor, has been used extensively as an anticonvulsant for more than 40 years 23. Much evidence confirms that VPA can induce the differentiation of many kinds of cancer cells *in vitro* and inhibit tumor invasion and metastasis *in vivo* 24, 25. VPA administration has been shown to delay the incidence of urinary bladder tumors in a mouse model 26. Because, epigenetic alternations are closely related to the development and progression of bladder cancer, VPA treatment could be a promising method to fight bladder cancer.

Since both melatonin and VPA exhibit promise as a single agent for numerous cancer cells, the combination of melatonin with VPA is expected to have a synergistic effect. Thus, the present study aimed to investigate the synergistic effects of melatonin in combination with VPA on the inhibition of cell growth and the induction of cell death in bladder cancer cells. Understanding the underlying mechanisms by which the agents induce cell death important to the translation of knowledge into application in the clinics. There are several mechanism associated with cellular death, including apoptosis, autophagy and necrosis. Apoptosis is considered a programmed form of cell death. Apoptotic triggers induce the synthesis or activation of proapoptotic Bcl‐2 homology region 3 (BH3)‐only proteins, including HRK (also known as DP5) 27. Apoptosis is divided into intrinsic and extrinsic pathways, and the extrinsic signaling pathways involve transmembrane receptor‐mediated interactions 28. Apoptotic or survival signals are the consequence of the death receptor family, members of which include Fas cell surface death receptor (FAS), TNF‐receptor superfamily member 10A (TNFRSF10A, also known as DR4) and TNF‐receptor superfamily member 10B (TNFRSF10B, also known as DR5) being activated by death ligands 29. Cells receive external apoptotic signals through FAS, a member of the TNF‐receptor superfamily 30. An alternative death pathway, autophagy is a catabolic process which is important for cellular homeostasis by controlling the turnover of cytoplasmic constituents 31. Autophagy‐related genes (ATGs) are critical to the process of autophagy. Among these ATG proteins, the soluble cytosolic form of microtubule‐associated protein 1 light chain 3 (LC3‐I) is conjugated to phosphatidylethanol‐amine to generate LC3‐II during the formation of autophagosomes 32. Thus, the LC3‐II/LC3‐I ratio is regarded as a common measure of autophagic activity 33. In contrast to apoptosis and autophagy, necrosis was regarded as random and passive cell death without definable mediators in earlier work. In recent years, many programmed models of necrosis have been identified. Necroptosis, triggered by death receptors, requires the receptor‐activated protein kinase (RIPK)3‐dependent phosphorylation of mixed lineage kinase domain‐like (MLKL) to induce plasma membrane pore formation 34. RIPK1 possesses kinase‐dependent and scaffolding functions which could either inhibit or trigger necroptosis and apoptosis 35. Parthanatos, another model of regulated necrosis, reflects cell death related to poly (ADP‐ribose) polymerase 1 (PARP‐1) overactivation 36. Furthermore, endoplasmic reticulum (ER)‐stress leads to the induction of autophagy, which in turn, trigger cell survival or death depending on the situation 37. Many researches have indicated that ER‐stress induction is closely related to the process of epithelial–mesenchymal transition (EMT) 38, 39. Therefore, the present study also compared the effects of melatonin and VPA on ER‐stress induction and EMT process in bladder cancer cells.

## Materials and methods

### Cell lines and chemicals

Human bladder cancer cells UC3 was maintained in high‐glucose DMEM containing 10% fetal bovine serum and 1% penicillin‐streptomycin, and incubated at 37 °C in a humidified incubator with 5% CO_2_. Melatonin and VPA were purchased from Sigma Chemical Co. (St. Louis, MO, USA).

### Drug treatment

Melatonin was dissolved in dimethyl sulfoxide and prepared as a stock solution at 10^−1^ m. The stock solution was stored at −20 °C. The stock solution was added to culture medium at different concentrations according to different experimental procedures.

The concentrated stock solution of VPA was made at 1 m in sterile water and stored at −20 °C. The VPA stock solution was dissolved into the cell culture medium at different concentrations according to different experimental procedures.

Before treatment, the UC3 cells were seeded onto 100 mm cell culture dishes at a density of 1 × 10^6^ cells/dish and incubated at 37 °C in a humidified incubator with 5% CO_2_.

### Cell viability assays

Cell viability was determined via quantitative colorimetric crystal violet staining, MTT and LDH assays following treatment with melatonin, VPA and combination therapy with both. In these assays, cells were seeded (15 × 10^4^ cells·mL^−1^) in 100 μL medium/well in 96‐well plates, incubated overnight, and treated with various concentrations of the indicated compounds. All the analyses were performed three times.

Cytotoxicity of a drug was measured via the absorbance of the crystal violet stained cells. After a 24 h‐exposure to melatonin, VPA or combination of the two, 10% methanol (100 μL) was added and incubated for 30 s at room temperature. After discarding the methanol, 0.1% crystal violet (100 μL) was added and the cells were incubated for 20 min at room temperature. Crystal violet was discarded and the plate was rinsed under running tap water, then left to dry. Next, 33% acetic acid (100 μL) was added to the wells and samples were incubated for 30 min at room temperature. After incubation, absorbance was read using an ELISA plate reader at 570 nm.

The MTT assay was also applied to examine the viability of UC3 cells following treatment at different time points. Briefly, the media were replaced by 100 μL MTT and incubation was continued for 4 h. Then, dimethyl sulfoxide (DMSO) was used to dissolve the formazan crystals formed by MTT. Optical absorbance was determined at 490 nm using an ELISA plate reader.

For the LDH assay, the LDH Cytotoxicity Assay Kit (Beyotime, Hangzhou, China) was used. At the end of drug treatment, the culture medium was discarded. LDH release solution (150 μL) was added into each well and incubated at 37 °C in a humidified incubator with 5% CO_2_ for 1 h. LDH incubation medium (120 μL) was collected from each well and added to a new 96‐well plate. LDH assay solution (60 μL) was added in to each well and samples were incubated for 30 min in the dark. The LDH activity was measured using a microplate reader at 490 nm following the manufacturer's instructions.

### Quantification of apoptosis by flow cytometry

Apoptosis was analyzed using Annexin V‐FITC/PI kit (BD Biosciences, San Jose, CA, USA). Cells were treated with melatonin, VPA or the combination of the two for 24 h and harvested using trypsin‐EDTA. Cells were washed with rinse solution. Staining with Annexin V‐FITC and PI were performed following the manufacturer's instruction. Data were analyzed using flow cytometry (Fortessa, BD Biosciences).

### Cell‐cycle distribution analysis by flow cytometry

The cell‐cycle distribution analysis was determined via propidium iodide (PI) staining. After treatment with melatonin, VPA and combine therapy with both for 24 h, cells were harvested with trypsin‐EDTA. After being washed with PBS, cells were fixed with 80% ethanol. Before staining, the cells were then washed twice with cold PBS and centrifuged at 800 ***g*** for 5 min. The pellet was stained with PI/RNase Staining Buffer Solution (FACScan, BD Biosciences) for 15 min in the dark. DNA content was examined by flow cytometry (Fortessa, BD Biosciences) and analyzed by modfit software (Verity Software House, Topsham, ME, USA).

### Real‐time quantitative PCR

Total RNA was extracted using TRIzol reagent (Invitrogen, Carlsbad, CA, USA), after which cDNA was synthesized with the All‐in‐One cDNA Synthesis SuperMix (Bimake, Houston, TX, USA) according to the manufacture's protocol. The PCR was performed according to the instructions of 2 × SYBR Green qPCR Master Mix (Bimake). Following initial denaturation at 95 °C for 15 min, the amplification conditions were as follows: 45 cycles of denaturation at 95 °C for 10 s, annealing at 60 °C for 30 s, and elongation at 72 °C for 20 s.

### Western blotting and protein quantification

Total protein was extracted from the cells with RIPA buffer. Protein concentrations were estimated using a BCA Protein Assay kit (Beyotime, Shanghai, China). Lysates were separated by 10% SDS/PAGE and transferred onto polyvinyl difluoride membranes (Millipore Inc., Billerica, MA, USA). Membranes were blocked in 5% skim milk solution and incubated overnight at 4 °C with primary antibodies. After being washed with Tris‐buffered saline containing 0.05% Tween‐20 (TBST) the next day, each membrane was incubated for 2 h with horseradish peroxidase (HRP)‐conjugated secondary antibodies at room temperature. The blots were visualized with an ECL system.

### Statistical analysis

Data were obtained from at least three independent experiments. The statistical significance of the differences between control and experimental data was analyzed using the ANOVA test. Differences were considered statistically significant when *P* values were less than 0.05.

## Results

### Effect of melatonin and VPA on bladder cancer cell survival

**Figure 1:**
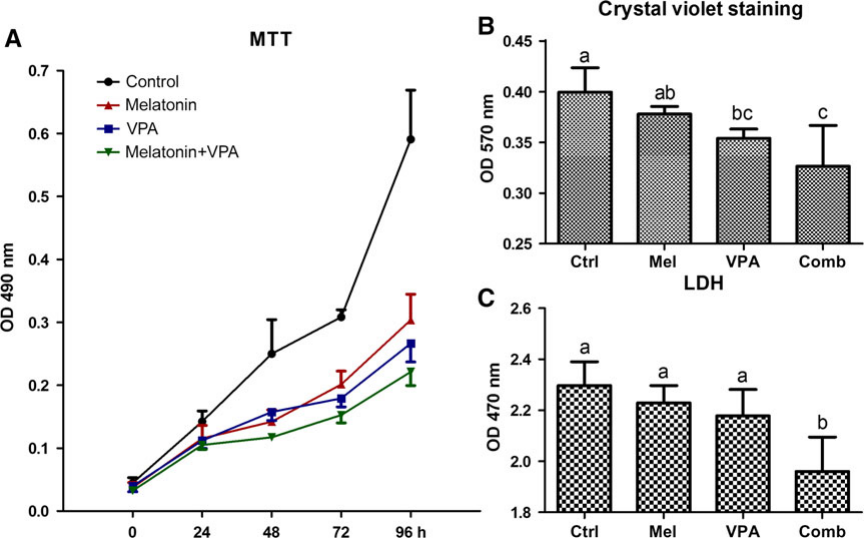
Cell viability of UC3 bladder cancer cells. (A) Cell proliferation and viability with melatonin (10^−6^ M) and/or VPA treatment (5 mM) were determined by an MTT assay at 0, 24, 48, 72 and 96 h. (B) Cytotoxicity among control (ctrl), melatonin (mel), VPA and combinatorial (comb) treated UC3 cells by crystal violet staining at 24 h. (C) LDH release assay among control (ctrl), melatonin (mel), VPA and combinatorial (comb) treated UC3 cells at 24 h. Error bars represent standard deviation. ^a,b,c^Values with different superscripts are significantly different.

To evaluate the combined effects of melatonin and VPA on bladder cancer cells, we performed cell viability assays on the bladder cancer cell line UC3. Cells were treated with 10^−6^ m melatonin and/or 5 mm VPA and then evaluated by MTT at 0, 24, 48, 72 and 96 h. The results indicated that the cell proliferation was significantly diminished in the presence of melatonin or VPA when compared with control cells from 24 to 96 h (Fig. 1A). Furthermore, the cell proliferation was further down‐regulated compared to individual treatment, when treated with a combination of melatonin and VPA.

Crystal violet assay was performed to confirm the influence of melatonin and/or VPA on the proliferation of bladder cancer cells. Treatment of UC3 cells with VPA for 24 h significantly reduced the viable cell number as compared to the control (*P* = 0.032; Fig. 1B). However, the viability of UC3 cells treated with melatonin for 24 h was not significantly reduced, as compared to the control cells (*P* = 0.276). Combination of both melatonin and VPA further reduced the viability of UC3 cells.

Analysis of the levels of lactate dehydrogenase (LDH) released into the culture media from dead/dying cells was performed to confirm the cytotoxicity with drug treatment. Compared with the control group, combination treatment with melatonin and VPA significantly reduced LDH leakage (*P* = 0.001; Fig. 1C). The results indicated that the cytotoxicity was enhanced with combination treatment.

**Figure 2:**
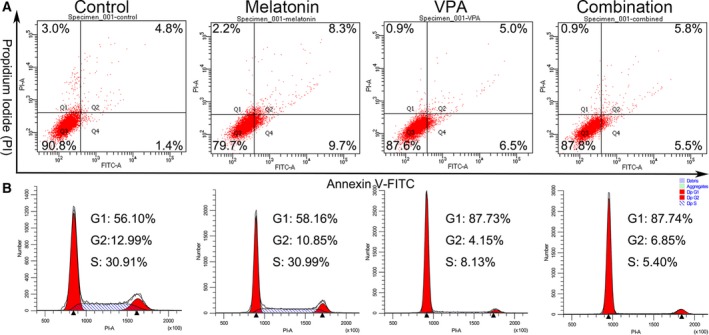
Apoptosis and cell‐cycle analysis of UC3 bladder cancer cells treated with melatonin and/or VPA for 24 h. (A) Cells were double‐stained with annexin V and propidium iodide (PI) and analyzed by flow cytometry. The gate settings distinguished living (bottom left), necrotic (top left), early apoptotic (bottom right) and late apoptotic (top right) cells. (B) Cells were stained with PI and the DNA content was analyzed by flow cytometry. G1, S and G2 indicate cell‐cycle phases.

Apoptosis was then determined by Annexin V‐FITC/PI method, which can distinguish healthy cells (Annexin V‐negative; PI‐negative) from early apoptotic (Annexin V‐positive; PI‐negative), late apoptotic (Annexin V‐positive; PI‐positive) and necrotic (Annexin V‐negative; PI‐positive) cells. The results showed that the apoptotic rate of UC3 cells with melatonin (18.0%), VPA (11.5%) and combinatorial treatment (11.3%) were higher than the control group (6.2%; Fig. 2A). However, there were no significant differences of the apoptotic rate between VPA and the combinatorial treatment.

To determine whether the combinatorial treatment would further alter cell‐cycle distribution, cell‐cycle analysis was also assessed by flow cytometry after PI staining. Results revealed that melatonin and VPA alone, and in combination, increased the fraction of cells in G0/G1 phase of the cell cycle (58.16%, 87.73% and 87.74% vs. 56.10%, respectively; Fig. 2B). Simultaneously, melatonin treatment reduced the proportion in the G2/M phase rather than the S phase when compared to the control. VPA treatment and combinatorial treatment reduced the proportion in both G2/M and S phase.

### Exploration of cell death by quantitative real‐time PCR

To determine the changes in some cell death‐related genes, gene expressions were evaluated in UC3 cells after treatment with 10^−6^ m melatonin and/or 5 mm VPA. In the present study, the gene analysis was performed to assess canonical cell death modes (apoptosis, necrosis and autophagy).

**Figure 3:**
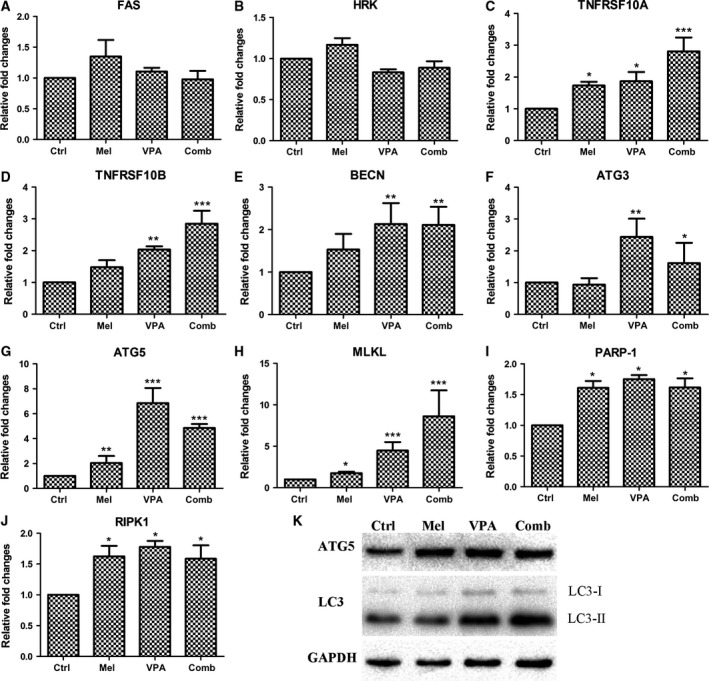
(A–J) Comparison of cell death‐related gene expression among UC3 cells with control (ctrl), melatonin (mel), VPA and combinatorial (comb) treatment by qPCR. (K) Gene expression was determined by western blotting. The data are presented as mean ± SD,* n* = 3. *P < 0.05 vs. control, **P ≤ 0.01 vs. control, and ***P ≤ 0.001 vs. control.

The expression of apoptosis‐related genes FAS, HRK, TNFRSF10A and TNFRSF10B was examined (Fig. 3). The expression of TNFRSF10A and TNFRSF10B was up‐regulated when UC3 cells were treated with melatonin or VPA (Fig. 3C,D). Furthermore, the combinatorial treatment with both melatonin and VPA further elevated the expression of TNFRSF10A and TNFRSF10B.

The expression of autophagy‐associated gene BECN, ATG3 and ATG5 was also investigated (Fig. 3). Treatment with melatonin did not significantly induce the BECN expression, but VPA stimulated the up‐regulation of BECN expression (Fig. 3E). In addition, the expression of BECN was also promoted by combinatorial treatment with melatonin and VPA. Similar to BECN expression, the expression of ATG3 was enhanced by VPA but not melatonin (Fig. 3F). Combinatorial treatment with melatonin and VPA also induced the up‐regulation of ATG3 expression. As for the expression of ATG5, both melatonin and VPA could significantly stimulate its expression (Fig. 3G). Surprisingly, the expression of ATG5 in response to the combinatorial treatment was higher than individual treatment with melatonin but lower than individual treatment with VPA. In addition, results of western blotting indicated that the expression of ATG5 protein was increased after drug treatment (Fig. 3K). However, the expression of ATG5 protein in cells with combinatorial treatment was lower than that of VPA treatment. The formation of LC3‐II/LC3‐I is a better reflection of autophagy induction than gene expression; therefore, the expression of LC3 was determined by western blotting. As shown in Fig. 3K, the ratio of LC3‐II/LC3‐I in the combinatorial treatment group was highest among these four groups.

Autophagy can modulate the outcome of necroptosis. In the present study, the expression of MLKL, PARP1 and RIPK1 was investigated (Fig. 3). The expression of all these genes was increased when compared to the control group (Fig. 3H–J). It is noteworthy that the expression of MLKL was obviously more increased in the VPA‐treated cells than in the melatonin‐treated cells (Fig. 3H). Furthermore, the expression of MLKL in UC3 cells with combinatorial treatment was significantly higher than cells with VPA treatment.

### Effects of melatonin and VPA on the expression of genes related to endoplasmic reticulum stress

**Figure 4:**
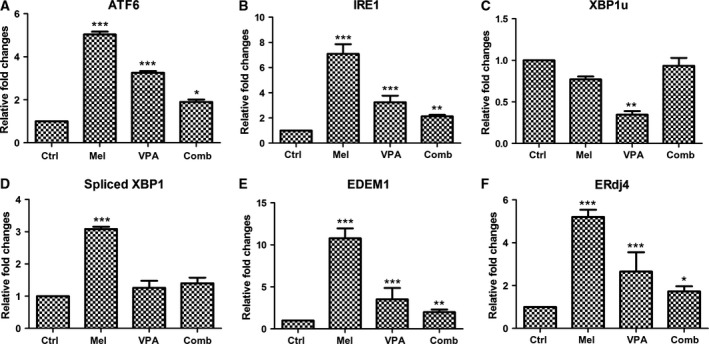
Expression of endoplasmic reticulum stress‐associated genes induced by melatonin and/or VPA treatment in UC3 bladder cancer cells. The data are presented as mean ± SD,* n* = 3. *P < 0.05 vs. control, **P ≤ 0.01 vs. control, and ***P ≤ 0.001 vs. control. Control (ctrl), melatonin (mel), and combinatorial treatment with both melatonin and VPA (comb).

To exploit the effects of 10^−6^ m melatonin and 5 mm VPA on ER‐stress, the expression of some ER‐stressor genes was determined (Fig. 4). The expression of ATF6 was promoted in UC3 with melatonin or VPA treatment, but was lower in VPA‐treated cells than in melatonin‐treated cells (Fig. 4A). In addition, the expression of ATF6 in UC3 cells with combinatorial treatment was lower than both melatonin and VPA treatment. Similar with the expression of ATF6, IRE1 was overexpressed in UC3 cells following melatonin, VPA or combinatorial treatment (Fig. 4B). The expression of IRE1 in combinatorial treatment cells was lowest among these three treatment groups. Activated IRE1 can direct the splicing of a 26‐nucleotide intron from XBP1u into a translational frameshift of XBP1 mRNA (spliced XBP1). Surprisingly, the expression of XBP1u in VPA‐treated cells was significantly lower than the control cells (Fig. 4C). There were similar expression level of XBP1u among the control, melatonin and combinatorial treatment groups. Spliced XBP1 was overexpressed just in the melatonin‐treated cells (Fig. 4D). It is well known that the spliced XBP1 would further enhance the expression of its target genes EDEM1 and ERdj4. Both EDEM1 and ERdj4 were overexpressed following melatonin, VPA or combinatorial treatment. Comparing individual treatment with melatonin or VPA, the expression of EDEM1 and ERdj4 in UC3 cells with combinatorial treatment was much lower than control (Fig. 4E,F). In conclusion, treatment with melatonin or VPA would result in the induction of gene expression related to ER‐stress in UC3 cells. However, combinatorial treatment could possibly reduce the extent of in the induction of gene expression related to ER‐stress.

### Combinatorial treatment further thwarts epithelial–mesenchymal transition

**Figure 5:**
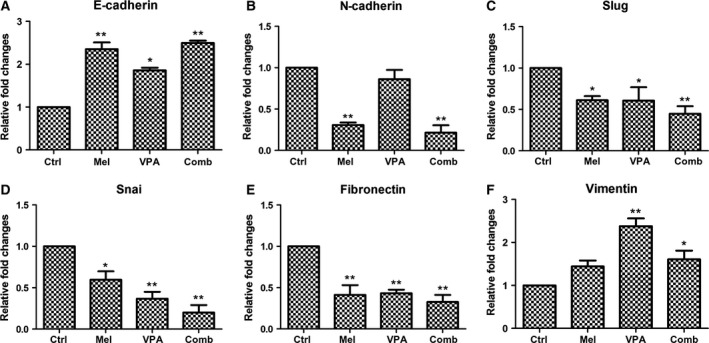
Exploration of epithelial–mesenchymal transition (EMT) at the gene level by quantitative real‐time PCR. The data are presented as mean ± SD,* n* = 3. *P < 0.05, as compared with the control group; **P ≤ 0.01, as compared with the control group. Control (ctrl), melatonin (mel), combinatorial treatment with both melatonin and VPA (comb).

The expression of some candidate transcription factors which promotes EMT was examined (Fig. 5). Comparing to the control cells, the expression of E‐cadherin in UC3 cells with 10^−6^ m melatonin, 5 mm VPA melatonin and combinatorial treatment of both was all enhanced (Fig. 5A). In contrast, the expression of *N*‐cadherin (Fig. 5B), Slug (Fig. 5C), Snail (Fig. 5D) and Fibronectin (Fig. 5E) was down‐regulated in UC3 cells following melatonin, VPA and combinatorial treatment. Furthermore, the expression of *N*‐cadherin, Slug, Snail and Fibronectin was lower in UC3 cells with combinatorial treatment than those cells with melatonin and VPA treatment. Surprisingly, the expression of Vimentin in UC3 cells with VPA and combinatorial treatment was higher than the control cells (Fig. 5F). Results of western blotting also confirmed that the expression of Vimentin protein was increased following melatonin, VPA and combinatorial treatment (Fig. 7F).

### Dissecting the signaling pathway following treatment

**Figure 6:**
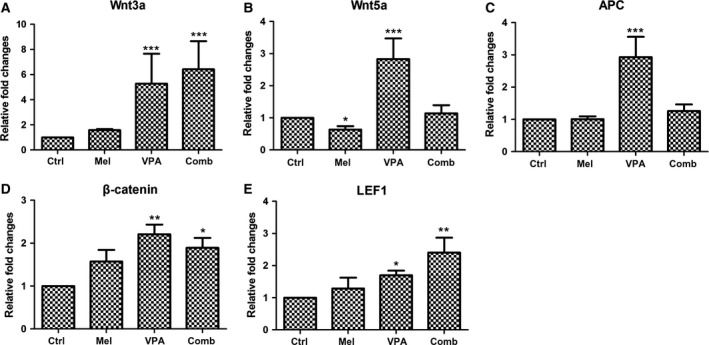
Expression of genes involved in Wnt signaling pathway. The data are presented as mean ± SD,* n* = 3. *P < 0.05 vs. control, **P ≤ 0.01 vs. control, and ***P ≤ 0.001 vs. control. Control (ctrl), melatonin (mel), combinatorial treatment with both melatonin and VPA (comb).

Several signaling pathways are involved in cancer occurrence and progression. In this study, the expression of Wnt and Raf/MEK/ERK signaling pathway molecules was investigated. As shown in Fig. 6A, the expression of Wnt3a was up‐regulated following VPA treatment. Though there were no significant differences on the expression of Wnt3a between control and melatonin treatment, the expression in UC3 cells with combinatorial treatment was highest among the four groups. Wnt5a was down‐regulated following melatonin treatment but up‐regulated following VPA treatment when compared with the combinatorial treatment (Fig. 6B). There were no significant differences in the expression of Wnt5a between control and combinatorial treatment cells. There were similar expression levels of adenomatous polyposis coli (APC) among control, melatonin and combinatorial treatment cells, but the expression of APC was significantly enhanced in VPA‐treated cells (Fig. 6C). The expression of β‐catenin (Fig. 6D) and Lef1 (Fig. 6D) was significantly promoted in UC3 cells following both VPA and combinatorial treatment. Generally speaking, the canonical Wnt signaling pathway was activated by VPA and combinatorial treatment.

**Figure 7:**
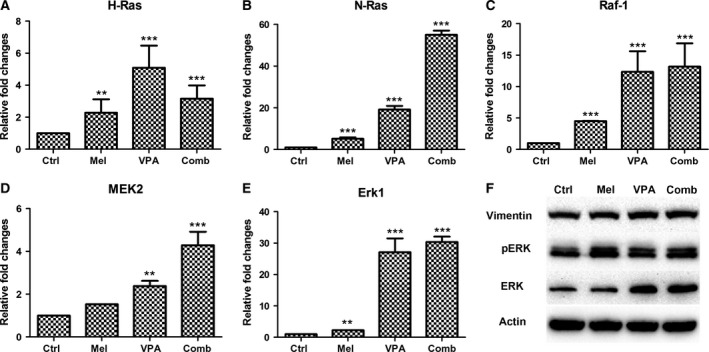
Expression of genes involved in Raf/MEK/ERK signaling pathway. (A–E) Gene expression was determined by quantitative real‐time PCR. (F) Gene expression was determined by western blotting. The data are presented as mean ± SD,* n* = 3. **P ≤ 0.01 vs. control, and ***P ≤ 0.001 vs. control. Control (ctrl), melatonin (mel) and combinatorial treatment with both melatonin and VPA (comb).

The potential involvement of the Raf/MEK/ERK signaling pathway in melatonin and/or VPA treatment was also determined. The expression of H‐ras, N‐ras, Raf1, MEK2 and Erk1 was examined by quantitative PCR. The results indicated the expression of all of these genes was increased following melatonin, VPA or combinatorial treatment (Fig. 7). Except for H‐ras, the expression of N‐ras, Raf1, MEK2 and Erk1 in UC3 cells with combinatorial treatment was even higher than the expression in the cells with VPA or melatonin treatment alone. These results were further confirmed by western blots for Erk1 protein, which revealed stronger bands in UC3 cells with VPA and combinatorial treatment than that in the control group (Fig. 7F). Furthermore, the phospho‐Erk1/2 (pErk1/2) protein levels were increased in UC3 cells following melatonin, VPA and combinatorial treatment (Fig. 7F). The observed changes in these genes examined may implicate the participation of Raf/MEK/ERK signaling pathway.

## Discussion

In the present study, the response of human bladder cancer cells UC3 to combinatorial melatonin and VPA treatment was determined. As expected, combinatorial treatment with melatonin and VPA effectively suppressed cell proliferation. To exploit the underlying mechanism, the expression of a panel of cell death‐related genes was evaluated. The expression of TNFRSF10A and TNFRSF10B was further enhanced by combinatorial treatment, suggesting that apoptosis‐related genes were involved in the regulation of proliferation of bladder cancer cells with melatonin and/or VPA.

Autophagy is widely accepted as a cytoprotective mechanism against neurodegenerative diseases, and various clinical interventions are moving forward to enhance autophagy as a therapeutic intervention 40. However, the role of autophagy in cancer is controversial due to both negative and positive effects in cancer treatment. Results of this study indicated that the gene expression patterns associated with autophagy induced by melatonin and VPA are different. For example, the expression of ATG3 and BECN was significantly increased by VPA treatment but not melatonin treatment. According to reports in the literature, autophagy can be enhanced 41, 42 or reduced 43, 44 by melatonin treatment, depending on the cell line. However, as far as we know, majority of the literature indicates that VPA induces autophagy 45, 46.

To further exploit the role of melatonin and/or VPA in inducing autophagy, necroptosis and ER‐stress were also analyzed in this study. Though the causal association between autophagy and various forms of regulated or nonregulated cell death remain elusive, increasing numbers of reports have shown that autophagy can modulate the outcome of necroptosis 47. The up‐regulated expression of MLKL, PARP1 and RIPK1 in the present study suggested that melatonin and VPA treatment would trigger the induction of necroptosis. Necroptosis is a form of programmed necrosis, which has recently emerged as a potential target for anticancer therapy 48. The expression of MLKL in UC3 cells with combinatorial treatment was significantly higher than cells with individual treatment of melatonin or VPA, indicating that combinatorial treatment with melatonin and VPA could be a potential cancer therapeutic. Besides necroptosis, autophagy is also associated with ER‐stress. ER‐stress has been posited as a potential anticancer target 49, and increasing evidence indicates that ER‐stress can trigger autophagy 50. The expression of IRE1, ATF6, EDEM1 and ERdj4 was shown to enhance by melatonin, VPA and combinatorial treatment, suggesting that ER‐stress was induced. However, the expression of spliced XBP1 was not promoted by VPA and combinatorial treatment (*P* > 0.05), possibly attributed to the decreasing expression of XBP1u.

Melatonin has been shown to effectively inhibit the EMT process 51. In addition, the attenuation of EMT signaling with melatonin treatment has been shown to associate with ER‐stress, considered a major molecular mechanism of melatonin anticancer activity 39. However, the role of VPA in the regulation of the EMT can be both positive and negative. For example, EMT is reversed by VPA by inhibiting the enhancement of invasion and metastasis in esophageal squamous cell carcinoma 52. VPA was also shown to enhance the EMT process via transcriptional and post‐transcriptional up‐regulation of Snail in hepatocarcinoma cells 53. In the present study, combinatorial treatment further decreased the expression of *N*‐cadherin, Slug, Snail and Fibronectin over individual melatonin or VPA treatment, suggesting that the EMT was possibly further inhibited by combinatorial treatment.

The Wnt and Raf/MEK/ERK signaling pathways are important for the development and progression of cancer. Previous study has revealed that β‐catenin can be activated by melatonin to protect neuronal cells through regulating antiapoptotic proteins 54. Many studies also document that the Wnt pathway can be activated by VPA exposure 55, 56. In the present study, expression of some Wnt signaling genes was increased following treatment, indicating that the Wnt signaling was possibly activated. In addition, the gene expression involved in Raf/MEK/ERK pathway was also enhanced by melatonin, VPA and combinatorial treatment. In fact, a handful of reports have shown the effects of melatonin and VPA on the activation of Raf/MEK/ERK pathway. Melatonin is effective in preventing the ischemic brain injury‐induced down‐regulation of Raf‐1, MEK1/2 and ERK1/2 phosphorylation 57, suggesting that the activation of Raf/MEK/ERK cascade can be mediated by Melatonin. In human hepatocytes, the ERK pathway can be activated by VPA exposure 58. Given that both melatonin and VPA have been reported to prevent tumor growth and progression in animal models of cancer 59, 60, it would be interesting to determine the combinatorial effects *in vivo* in our future studies.

Results of the present study reveal that combinatorial treatment was not superior to melatonin or VPA treatment alone in some cases. Combinatorial treatment did not exhibit synergy in inducing apoptosis in comparison to melatonin or VPA treatment alone. For one thing, combination of 10^−6^ m melatonin and 5 mm VPA may be a high dose, which may cause nonspecific cell death or lead to apoptotic cells more fragile to centrifuging during Annexin V/PI staining. For another thing, the status of gene expression, in turn, may explain why there was a lower apoptotic rate with combinatorial treatment than with melatonin treatment alone. For example, there were no significant differences in the expression of PARP‐1 and RIPK1 among melatonin, VPA and combinatory treatment groups. The expression of some ER‐stressors was not further modulated with combinatorial treatment over melatonin or VPA treatment alone. Further studies should focus on optimizing the concentration and duration of combinatorial treatment and determining the impacts on gene expression.

## Author contributions

SL, BL, HJ, YJ and ZP performed the experiments. YH developed the concept, designed the experiments and wrote the paper.

## References

[1] van Kessel KE , Zuiverloon TC , Alberts AR , Boormans JL and Zwarthoff EC (2015) Targeted therapies in bladder cancer: an overview of in vivo research. Nat Rev Urol 12, 681–694. 26390971 10.1038/nrurol.2015.231

[2] Xu XS , Wang L , Abrams J and Wang G (2011) Histone deacetylases (HDACs) in XPC gene silencing and bladder cancer. J Hematol Oncol 4, 17. 21507255 10.1186/1756-8722-4-17 PMC3108377

[3] Reiter RJ , Tan DX , Manchester LC and Qi W (2001) Biochemical reactivity of melatonin with reactive oxygen and nitrogen species: a review of the evidence. Cell Biochem Biophys 34, 237–256. 11898866 10.1385/CBB:34:2:237

[4] Mao L , Summers W , Xiang S , Yuan L , Dauchy RT , Reynolds A , Wren‐Dail MA , Pointer D , Frasch T , Blask DE *et al* (2016) Melatonin represses metastasis in Her2‐Postive human breast cancer cells by suppressing RSK2 expression. Mol Cancer Res 14, 1159–1169. 27535706 10.1158/1541-7786.MCR-16-0158 PMC5107120

[5] Zou DB , Wei X , Hu RL , Yang XP , Zou L , Zhang SM , Zhu HQ , Zhou Q , Gui SY and Wang Y (2015) Melatonin inhibits the migration of colon cancer RKO cells by down‐regulating myosin light chain kinase expression through cross‐talk with p38 MAPK. Asian Pac J Cancer Prev 16, 5835–5842. 26320459 10.7314/apjcp.2015.16.14.5835

[6] Akbarzadeh M , Nouri M , Banekohal MV , Cheraqhi O , Tajalli H , Movassaqhpour A , Soltani S , Cheraghi H , Feizy N , Montazersaheb S *et al* (2016) Effects of combination of melatonin and laser irradiation on ovarian cancer cells and endothelial lineage viability. Lasers Med Sci 31, 1565–1572. 27365110 10.1007/s10103-016-2016-6

[7] Erren TC , Slanger TE , Gross JV and Reiter RJ (2015) Melatonin, sleep, and prostate cancer in elderly men: study, hypothesis development, and icelandic options. Eur Urol 67, 195–197. 25282366 10.1016/j.eururo.2014.09.033

[8] Yun M , Kim EO , Lee D , Kim JH , Kim J , Lee H , Lee J and Kim SH (2014) Melatonin sensitizes H1975 non‐small‐cell lung cancer cells harboring a T790M‐targeted epidermal growth factor receptor mutation to the tyrosine kinase inhibitor gefitinib. Cell Physiol Biochem 34, 865–872. 25199820 10.1159/000366305

[9] Swarnakar S , Paul S , Singh LP and Reiter RJ (2011) Matrix metalloproteinases in health and disease: regulation by melatonin. J Pineal Res 50, 8–20. 20964709 10.1111/j.1600-079X.2010.00812.x

[10] Ordonez R , Carbajo‐Pescador S , Prieto‐Dominguez N , Garcia‐Palomo A , Gonzalez‐Gallego J and Mauriz JL (2014) Inhibition of matrix metalloproteinase‐9 and nuclear factor kappa B contribute to melatonin prevention of motility and invasiveness in HepG2 liver cancer cells. J Pineal Res 56, 20–30. 24117795 10.1111/jpi.12092

[11] Lin YW , Lee LM , Lee WJ , Chu CY , Tan P , Yang YC , Chen WY , Yang SF , Hsiao M and Chien MH (2016) Melatonin inhibits MMP‐9 transactivation and renal cell carcinoma metastasis by suppressing Akt‐MAPKs pathway and NF‐kappaB DNA‐binding activity. J Pineal Res 60, 277–290. 26732239 10.1111/jpi.12308

[12] Gonçalves Ndo N , Colombo J , Lopes JR , Gelaleti GB , Moschetta MG , Sonehara NM , Hellmén E , Zanon Cde F , Oliani SM and Zuccari DA (2016) Effect of melatonin in epithelial mesenchymal transition markers and invasive properties of breast cancer stem cells of canine and human cell lines. PLoS One 11, e0150407. 26934679 10.1371/journal.pone.0150407 PMC4774906

[13] Yeh CM , Lin CW , Yang JS , Yang WE , Su SC and Yang SF (2016) Melatonin inhibits TPA‐induced oral cancer cell migration by suppressing matrix metalloproteinase‐9 activation through the histone acetylation. Oncotarget 7, 21952–21967. 26980735 10.18632/oncotarget.8009 PMC5008336

[14] Pan Y and Niles LP (2015) Epigenetic mechanisms of melatonin action in human SH‐SY5Y neuroblastoma cells. Mol Cell Endocrinol 402, 57–63. 25578604 10.1016/j.mce.2015.01.003

[15] Yamanishi M , Narazaki H and Asano T (2015) Melatonin overcomes resistance to clofarabine in two leukemic cell lines by increased expression of deoxycytidine kinase. Exp Hematol 43, 207–214. 25461250 10.1016/j.exphem.2014.11.001

[16] Wei JY , Li WM , Zhou LL , Lu QN and He W (2015) Melatonin induces apoptosis of colorectal cancer cells through HDAC4 nuclear import mediated by CaMKII inactivation. J Pineal Res 58, 429–438. 25752481 10.1111/jpi.12226

[17] Chu J , Tu Y , Chen J , Tan D , Liu X and Pi R (2016) Effects of melatonin and its analogues on neural stem cells. Mol Cell Endocrinol 420, 169–179. 26499395 10.1016/j.mce.2015.10.012

[18] Sharma R , Ottenhof T , Rzeczkowska PA and Niles LP (2008) Epigenetic targets for melatonin: induction of histone H3 hyperacetylation and gene expression in C17.2 neural stem cells. J Pineal Res 45, 277–284. 18373554 10.1111/j.1600-079X.2008.00587.x

[19] Fan C , Pan Y , Yang Y , Di S , Jiang S , Ma Z , Li T , Zhang Z , Li W , Li X *et al* (2015) HDAC1 inhibition by melatonin leads to suppression of lung adenocarcinoma cells via induction of oxidative stress and activation of apoptotic pathways. J Pineal Res 59, 321–333. 26184924 10.1111/jpi.12261

[20] Vallo S , Xi W , Hudak L , Juengel E , Tsaur I , Wiesner C , Haferkamp A and Blaheta RA (2011) HDAC inhibition delays cell cycle progression of human bladder cancer cells in vitro. Anticancer Drugs 22, 1002–1009. 21822119 10.1097/CAD.0b013e32834a2c70

[21] Wang D , Jing Y , Ouyang S , Liu B , Zhu T , Niu H and Tian Y (2013) Inhibitory effect of valproic acid on bladder cancer in combination with chemotherapeutic agents in vitro and in vivo. Oncol Lett 6, 1492–1498. 24179547 10.3892/ol.2013.1565 PMC3813788

[22] Mawatari T , Ninomiya I , Inokuchi M , Harada S , Hayashi H , Oyama K , Makino I , Nakagawara H , Miyashita T , Tajima H *et al* (2015) Valproic acid inhibits proliferation of HER2‐expressing breast cancer cells by inducing cell cycle arrest and apoptosis through Hsp70 acetylation. Int J Oncol 47, 2073–2081. 26497673 10.3892/ijo.2015.3213 PMC4665753

[23] van Breemen MS , Rijsman RM , Taphoorn MJ , Walchenbach R , Zwinkels H and Vecht CJ (2009) Efficacy of anti‐epileptic drugs in patients with gliomas and seizures. J Neurol 256, 1519–1526. 19434440 10.1007/s00415-009-5156-9

[24] Göttlicher M , Minucci S , Zhu P , Krämer OH , Schimpf A , Giavara S , Sleeman JP , Lo Coco F , Nervi C , Pelicci PG *et al* (2001) Valproic acid defines a novel class of HDAC inhibitors inducing differentiation of transformed cells. EMBO J 20, 6969–6978. 11742974 10.1093/emboj/20.24.6969 PMC125788

[25] Berendsen S , Broekman M , Seute T , Snijders T , van Es C , de Vos F , Regli L and Robe P (2012) Valproic acid for the treatment of malignant gliomas: review of the preclinical rationale and published clinical results. Expert Opin Investig Drugs 21, 1391–1415. 10.1517/13543784.2012.694425 22668241

[26] Ozawa A , Tanji N , Kikugawa T , Sasaki T , Yanagihara Y , Miura N and Yokoyama M (2010) Inhibition of bladder tumour growth by histone deacetylase inhibitor. BJU Int 105, 1181–1186. 19681894 10.1111/j.1464-410X.2009.08795.x

[27] Hockings C , Anwari K , Ninnis RL , Brouwer J , O'Hely M , Evangelista M , Hinds MG , Czabotar PE , Lee EF , Fairlie WD *et al* (2015) Bid chimeras indicate that most BH3‐only proteins can directly activate Bak and Bax, and show no preference for Bak versus Bax. Cell Death Dis 6, e1735. 25906158 10.1038/cddis.2015.105 PMC4650538

[28] Teocchi MA and D'Souza‐Li L (2016) Apoptosis through death receptors in temporal lobe epilepsy‐associated hippocampal sclerosis. Mediators Inflamm 2016, 8290562. 27006531 10.1155/2016/8290562 PMC4781997

[29] Lavrik I , Golks A and Krammer PH (2005) Death receptor signaling. J Cell Sci 118, 265–267. 15654015 10.1242/jcs.01610

[30] Li Q , Ching AK , Chan BC , Chow SK , Lim PL , Ho TC , Ip WK , Wongn CK , Lam CW Lee KK *et al* (2004) A death receptor‐associated anti‐apoptotic protein, BRE, inhibits mitochondrial apoptotic pathway. J Biol Chem 279, 52106–52116. 15465831 10.1074/jbc.M408678200

[31] Wang J , Liu Y , Li XH , Zeng XC , Li J , Zhou J , Xiao B and Hu K (2016) Curcumin protects neuronal cells against status epilepticus‐induced hippocampal damage through induction of autophagy and inhibition of necroptosis. Can J Physiol Pharmacol 9, 1–9. 10.1139/cjpp-2016-0154 28177687

[32] Tanida I , Ueno T and Kominami E (2008) LC3 and autophagy. Methods Mol Biol 445, 77–88. 18425443 10.1007/978-1-59745-157-4_4

[33] Chen Y , Zhou X , Qiao J and Bao A (2017) Autophagy is a regulator of TRAIL‐induced apoptosis in NSCLC A549 cells. J Cell Commun Signal 18, 1–8. 10.1007/s12079-016-0364-4 PMC5559391 28101818

[34] Chen X , Li W , Ren J , Huang D , He WT , Song Y , Yang C , Li W , Zheng X , Chen P *et al* (2014) Translocation of mixed lineage kinase domain‐like protein to plasma membrane leads to necrotic cell death. Cell Res 24, 105–121. 24366341 10.1038/cr.2013.171 PMC3879712

[35] Dannappel M , Vlantis K , Kumari S , Polykratis A , Kim C , Wachsmuth L , Eftychi C , Lin J , Corona T , Hermance N *et al* (2014) RIPK1 maintains epithelial homeostasis by inhibiting apoptosis and necroptosis. Nature 513, 90–94. 25132550 10.1038/nature13608 PMC4206266

[36] Andrabi SA , Kim NS , Yu SW , Wang H , Koh DW , Sasaki M , Klaus JA , Otsuka T , Zhang Z , Koehler RC *et al* (2006) Poly(ADP‐ribose) (PAR) polymer is a death signal. Proc Natl Acad Sci USA 103, 18308–18313. 17116882 10.1073/pnas.0606526103 PMC1838747

[37] Fernández A , Ordóñez R , Reiter RJ , González‐Gallego J and Mauriz JL (2015) Melatonin and endoplasmic reticulum stress relation to autophagy and apoptosis. J Pineal Res 59, 292–307. 26201382 10.1111/jpi.12264

[38] Shah PP , Dupre TV , Siskind LJ and Beverly LJ (2017) Common cytotoxic chemotherapeutics induce epithelial‐mesenchymal transition (EMT) downstream of ER stress. Oncotarget 8, 22625–22639. 28186986 10.18632/oncotarget.15150 PMC5410250

[39] Wu SM , Lin WY , Shen CC , Pan HC , Keh‐Bin W , Chen YC , Jan YJ , Lai DW , Tang SC , Tien HR *et al* (2016) Melatonin set out to ER stress signaling thwarts epithelial mesenchymal transition and peritoneal dissemination via calpain‐mediated C/EBPbeta and NFkappaB cleavage. J Pineal Res 60, 142–154. 26514342 10.1111/jpi.12295

[40] Towers CG and Thorburn A (2016) Therapeutic targeting of autophagy. EBioMedicine 14, 15–23. 28029600 10.1016/j.ebiom.2016.10.034 PMC5161418

[41] Ding K , Xu J , Wang H , Zhang L , Wu Y and Li T (2015) Melatonin protects the brain from apoptosis by enhancement of autophagy after traumatic brain injury in mice. Neurochem Int 91, 46–54. 26527380 10.1016/j.neuint.2015.10.008

[42] Chen Y , Zhang J , Zhao Q , Chen Q , Sun Y , Jin Y and Wu J (2016) Melatonin induces anti‐inflammatory effects to play a protective role via endoplasmic reticulum stress in acute pancreatitis. Cell Physiol Biochem 40, 1094–1104. 27960163 10.1159/000453164

[43] Yoo YM , Han TY and Kim HS (2016) Melatonin suppresses autophagy induced by clinostat in preosteoblast MC3T3‐E1 cells. Int J Mol Sci 17, 526. 27070587 10.3390/ijms17040526 PMC4848982

[44] de Luxán‐Delgado B , Potes Y , Rubio‐González A , Caballero B , Solano JJ , Fernández‐Fernández M , Bermúdez M , Rodrigues Moreira Guimarães M , Vega‐Naredo I , Boga JA *et al* (2016) Melatonin reduces endoplasmic reticulum stress and autophagy in liver of leptin‐deficient mice. J Pineal Res 61, 108–123. 27090356 10.1111/jpi.12333

[45] Zhang Y , Wu JY , Weng LH , Li XX , Yu LJ and Xu Y (2016) Valproic acid protects against MPP+‐mediated neurotoxicity in SH‐SY5Y cells through autophagy. Neurosci Lett 638, 60–68. 27956235 10.1016/j.neulet.2016.12.017

[46] Xia Q , Zheng Y , Jiang W , Huang Z , Wang M , Rodriguez R and Jin X (2016) Valproic acid induces autophagy by suppressing the Akt/mTOR pathway in human prostate cancer cells. Oncol Lett 12, 1826–1832. 27588130 10.3892/ol.2016.4880 PMC4998110

[47] Ryter SW , Mizumura K and Choi AM (2014) The impact of autophagy on cell death modalities. Int J Cell Biol 2014, 502676. 24639873 10.1155/2014/502676 PMC3932252

[48] Lalaoui N and Brumatti G (2016) Relevance of necroptosis in cancer. Immunol Cell Biol 95, 137–145. 27922620 10.1038/icb.2016.120

[49] Nieto‐Miguel T , Fonteriz RI , Vay L , Gajate C , Lopez‐Hernandez S and Mollinedo F (2007) Endoplasmic reticulum stress in the proapoptotic action of edelfosine in solid tumor cells. Cancer Res 67, 10368–10378. 17974980 10.1158/0008-5472.CAN-07-0278

[50] Nie T , Yang S , Ma H , Zhang L , Lu F , Tao K , Wang R , Yang R , Huang L , Mao Z *et al* (2016) Regulation of ER stress‐induced autophagy by GSK3beta‐TIP60‐ULK1 pathway. Cell Death Dis 7, e2563. 28032867 10.1038/cddis.2016.423 PMC5260977

[51] Yu N , Sun YT , Su XM , He M , Dai B and Kang J (2016) Melatonin attenuates TGFbeta1‐induced epithelial‐mesenchymal transition in lung alveolar epithelial cells. Mol Med Rep 14, 5567–5572. 27878256 10.3892/mmr.2016.5950

[52] Kanamoto A , Ninomiya I , Harada S , Tsukada T , Okamoto K , Nakanuma S , Sakai S , Makino I , Kinoshita J , Hayashi H *et al* (2016) Valproic acid inhibits irradiation‐induced epithelial‐mesenchymal transition and stem cell‐like characteristics in esophageal squamous cell carcinoma. Int J Oncol 49, 1859–1869. 27826618 10.3892/ijo.2016.3712 PMC5063503

[53] Wu L , Feng H , Hu J , Tian X and Zhang C (2016) Valproic acid (VPA) promotes the epithelial mesenchymal transition of hepatocarcinoma cells via transcriptional and post‐transcriptional up regulation of Snail. Biomed Pharmacother 84, 1029–1035. 27768928 10.1016/j.biopha.2016.10.023

[54] Jeong JK , Lee JH , Moon JH , Lee YJ and Park SY (2014) Melatonin‐mediated beta‐catenin activation protects neuron cells against prion protein‐induced neurotoxicity. J Pineal Res 57, 427–434. 25251028 10.1111/jpi.12182

[55] Qin L , Dai X and Yin Y (2016) Valproic acid exposure sequentially activates Wnt and mTOR pathways in rats. Mol Cell Neurosci 75, 27–35. 27343825 10.1016/j.mcn.2016.06.004

[56] Wang L , Liu Y , Li S , Long ZY and Wu YM (2015) Wnt signaling pathway participates in valproic acid‐induced neuronal differentiation of neural stem cells. Int J Clin Exp Pathol 8, 578–585. 25755748 PMC4348902

[57] Koh PO (2008) Melatonin attenuates the cerebral ischemic injury via the MEK/ERK/p90RSK/bad signaling cascade. J Vet Med Sci 70, 1219–1223. 19057141 10.1292/jvms.70.1219

[58] Bitman M , Vrzal R , Dvorak Z and Pavek P (2014) Valproate activates ERK signaling pathway in primary human hepatocytes. Biomed Pap Med Fac Univ Palacky Olomouc Czech Repub 158, 39–43. 23073524 10.5507/bp.2012.038

[59] Paroni R , Terraneo L , Bonomini F , Finati E , Virgili E , Bianciardi P , Favero G , Fraschini F , Reiter RJ , Rezzani R *et al* (2014) Antitumour activity of melatonin in a mouse model of human prostate cancer: relationship with hypoxia signalling. J Pineal Res 57, 43–52. 24786921 10.1111/jpi.12142

[60] Lee DH , Nam JY , Chang Y , Cho H , Kang SH , Cho YY , Cho E , Lee JH , Yu SJ , Kim YJ *et al* (2017) Synergistic effect of cytokine‐induced killer cell with valproate inhibits growth of hepatocellular carcinoma cell in a mouse model. Cancer Biol Ther 18, 67–75. 28055304 10.1080/15384047.2016.1276132 PMC5323016

